# Expression Analysis of *PIN* Genes in Root Tips and Nodules of *Medicago truncatula*

**DOI:** 10.3390/ijms17081197

**Published:** 2016-07-25

**Authors:** Izabela Sańko-Sawczenko, Barbara Łotocka, Weronika Czarnocka

**Affiliations:** Department of Botany, Faculty of Agriculture and Biology, Warsaw University of Life Sciences—SGGW, Nowoursynowska 159, 02-776 Warsaw, Poland; izabela_sanko_sawczenko@sggw.pl (I.S.-S.); barbara_lotocka@sggw.pl (B.Ł.)

**Keywords:** *Medicago truncatula*, root nodule, auxin, PIN

## Abstract

Polar auxin transport is dependent on the family of PIN-formed proteins (PINs), which are membrane transporters of anionic indole-3-acetic acid (IAA^−^). It is assumed that polar auxin transport may be essential in the development and meristematic activity maintenance of *Medicago truncatula* (*M. truncatula*) root nodules. However, little is known about the involvement of specific PIN proteins in *M. truncatula* nodulation. Using real-time quantitative PCR, we analyzed the expression patterns of all previously identified *MtPIN* genes and compared them between root nodules and root tips of *M. truncatula*. Our results demonstrated significant differences in the expression level of all 11 genes (*MtPIN1*–*MtPIN11*) between examined organs. Interestingly, *MtPIN9* was the only *PIN* gene with higher expression level in root nodules compared to root tips. This result is the first indication of PIN9 transporter potential involvement in *M. truncatula* nodulation. Moreover, relatively high expression level in root nodules was attributed to *MtPINs* encoding orthologs of *Arabidopsis thaliana* PIN5 subclade. PIN proteins from this subclade have been found to localize in the endoplasmic reticulum, which may indicate that the development and meristematic activity maintenance of *M. truncatula* root nodules is associated with intracellular homeostasis of auxins level and their metabolism in the endoplasmic reticulum.

## 1. Introduction

Nitrogen is the primary and most important nutrient for plants, since it is an element for amino acids and nucleobases biosynthesis. Its availability in soil is frequently a major limiting factor for plant growth and, consequently, crop yield. The Earth’s atmosphere is a rich source of dinitrogen, but, unfortunately, plants cannot directly assimilate it in this form. However, plant species from the Fabaceae family have evolved an ability of establishing symbiosis with nitrogen-fixing bacteria, collectively called rhizobia, which allows them to exploit atmospheric nitrogen sources. This type of symbiosis is an effective evolutionary adaptation, which enables fabaceans to accumulate high levels of nitrogen in their tissues [1].

*Medicago truncatula* is a model fabacean species in the studies of plant interactions with rhizobia. The symbiosis initiates with a molecular dialogue in the rhizosphere between microorganisms and plant’s roots. Such molecular communication enables rhizobia to find their compatible host plant. Flavonoids, which are secreted by fabacean roots, induce bacterial Nod factors synthesis in rhizobia [2]. The recognition of specific Nod factors triggers root hair deformation relying on their curling around microsymbionts’ cells [3]. Subsequently, rhizobia induce local plant cell wall rebuilding, which gives rise to the infection thread, allowing bacteria the colonization of host tissues. At the same time, the nodule primordium starts to develop. When the infection thread reaches the nodule primordium, rhizobia are endocytosed by host cells. Each bacterial cell is surrounded by a peribacteroid membrane originating from the infection thread’s plasma membrane. This structure is called the symbiosome, and the bacteria located inside—bacteroid [4]. In bacteroids, metabolism shifts from the processes specific for free-living bacteria to increased activity of proteins associated with dinitrogen fixation and ATP synthesis, which is crucial for dinitrogen reduction [5]. Host plants provide to bacteroids the energy source and metabolites essential for dinitrogen fixation. The main carbon sources supplied are C4-dicarboxylic acids such as malate, which are intermediates in the citric acid cycle [6]. Furthermore, the host plant needs to ensure a microaerobic environment in the infected cells. This is indispensable for the proper activity of nitrogenase, an oxygen-labile enzyme directly responsible for the dinitrogen fixation. Such an environment is created by the presence of symbiotic leghemoglobins, hemoproteins present in root nodules. Like human hemoglobin, they have strong affinity towards oxygen, and thus effectively decrease their concentration in nodule tissues [7]. In symbiosomes, dinitrogen is converted into ammonium and subsequently secreted for the uptake by plant tissues. Since ammonium is toxic to plant cells, it is immediately assimilated by presumably three interdependent metabolic pathways engaging asparagine synthetase, glutamine synthetase or glutamate dehydrogenase [8].

A mature *Medicago truncatula* (*M. truncatula*) root nodule has several distinct zones. From the apical part of the nodule, the following developmental zones can be distinguished: (I) the rhizobial-free meristematic zone, providing indeterminate nodule growth; (II) the infection zone, in which bacterial cells are released from the infection threads and cell differentiation begins; (II/III) the interzone, usually consisting of one to three layers of cells that accumulate starch rapidly; (III) the dinitrogen-fixing zone with mature bacteroids; (IV) the senescence zone, which contains degenerated symbiosomes [9]; and (V) the saprophytic zone with rod-shaped rhizobia colonizing degraded host’s cells [10]. The nodule meristem—together with the bacteroid-containing tissue are surrounded with lateral nodule tissues—an inner cortex with vascular bundles, a nodule endodermis and an outer cortex [9].

An essential role of auxins (principally indole-3-acetic acid—IAA) in nodulation has been assumed for a long time. These plant hormones are involved in numerous, sometimes very divergent physiological processes, such as embryogenesis, organogenesis, plant tropisms, maintenance of meristematic activity, differentiation of vascular tissues, root elongation, fruit development, apical dominance and responses to many environmental stimuli. This phenomenon can be explained by the ability of auxins to be directionally transported and accumulated in particular cells and tissues. Auxins are synthesized in young apical tissues and can be transported by two different pathways: by phloem parenchyma cells (non-polar transport) or by cell-to-cell transport as a result of polar auxin transport (PAT). The second pathway is crucial for the local accumulation of these hormones and is dependent on polar auxin transporters such as auxin efflux carriers—PIN-formed proteins (PINs), PGP proteins that work both as influx and efflux carriers, as well as auxin influx carriers—AUX1/LAX [11]. The protonated form of IAA (IAAH) has the ability to penetrate cell membranes in accordance with the concentration gradient. In cytosolic environments (pH = 7), IAAH dissociates into IAA^–^ and H^+^. Deprotonated IAA^−^ is trapped inside the cell and cannot passively diffuse through the plasma membrane. Thus, auxin efflux carriers (PINs) are needed to transport IAA^–^ from the cell [11,12,13].

PIN proteins are polar carriers, which means that their location in the plasma membrane of transport-competent cells is asymmetric. They are predominantly positioned on only one side of the cell, which empowers required direction of auxin transport [13]. However, in *Arabidopsis thaliana* (*A.* *thaliana*) cells, PIN proteins have also been found to localize in endoplasmic reticulum (ER) membranes. AtPIN5, AtPIN6 and AtPIN8, belonging to PIN5 subclade, mediate auxins transport from cytosol to ER lumen [14,15,16,17].

Eleven genes encoding PIN proteins have been characterized in *M. truncatula*: *MtPIN1*–*MtPIN11* [18,19]. Insightful phylogenetic analyses of genomic and protein sequences of MtPINs revealed conserved N- and C-terminal regions of transmembrane domains and variable middle region of cytoplasmic domain. Evolutionary relations and possible origins of MtPINs have also been explained, which enabled identification of *A. thaliana* and *M. truncatula* probable orthologs [18,19].

The subcellular localization of PIN proteins, their mode of action, and involvement in specific molecular pathways are well-known in *A. thaliana*. However, our knowledge concerning PIN proteins in *M. truncatula*, especially their function in the nodulation process, is still very limited. Although there are some data indicating the role of auxins in the nodulation [20,21,22,23,24,25], and there is a huge gap regarding detailed analysis of PIN involvement in nodule formation and maintenance of their meristematic activity. In this study, we performed a comprehensive analysis of *PIN* expression, by examining their transcription levels in *M. truncatula* root tips and nodules. Our results indicate the importance of polar auxins transporters in the nodulation. Based on collected data, we identified these auxin transporters, which may play a crucial role in the nodulation. Our study is the first such detailed one concerning expression of PIN-encoding genes in the model species for rhizobium-fabacean symbiosis—*M. truncatula*.

## 2. Results

To perform a detailed expression analysis of *PIN* genes in *M. truncatula* root tips and nodules, we employed the real-time quantitative PCR (qPCR) technique. Primers were designed for 11 *MtPINs* identified previously [18,19]. In order to unambiguously identify the phylogenetic relationship between *M. truncatula* and *A. thaliana* orthologs, we performed a BLAST alignment of each MtPIN’s coding DNA sequence (CDS) and full protein sequence with the *A. thaliana* nucleotide or protein database, respectively. On the basis of this comparison, we identified *A. thaliana* orthologs that are most closely related to the corresponding MtPINs (Table 1). Our results proved some minor differences between previously identified *A. thaliana* and *M. truncatula* orthologs [18,19] and our data. For instance, based on protein alignment, MtPIN7 appeared to be most closely related to AtPIN7, not AtPIN2, as previously described [18,19].

**Figure 1:**
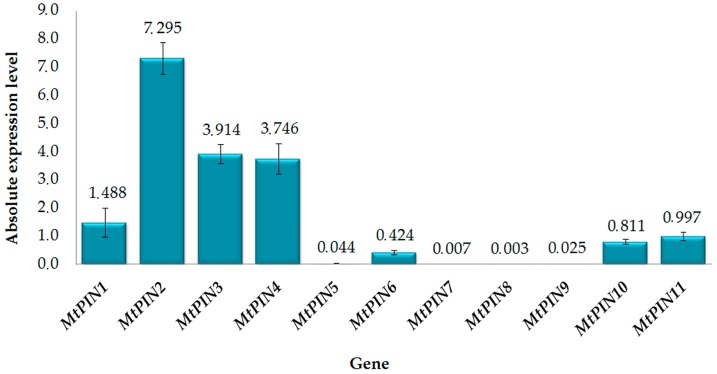
Absolute, normalized level of *PIN* expression in *M. truncatula* root tips. Mean values (±SE) are derived from two biological replicates, for which three individual qPCR reactions were performed (*n* = 6). Expression level for each *PIN* was normalized to the endogenous control.

Absolute, normalized level of *MtPIN*s expression in root tips is presented in Figure 1. Results revealed that all 11 *MtPIN*s were expressed in root tips. However, their expression level differed remarkably. *MtPIN1* to *MtPIN4* had significantly higher expression level than the other seven *MtPIN*s. The highest expression was found for *MtPIN2*, whereas the lowest was for *MtPIN7* and *MtPIN8*.

**Figure 2:**
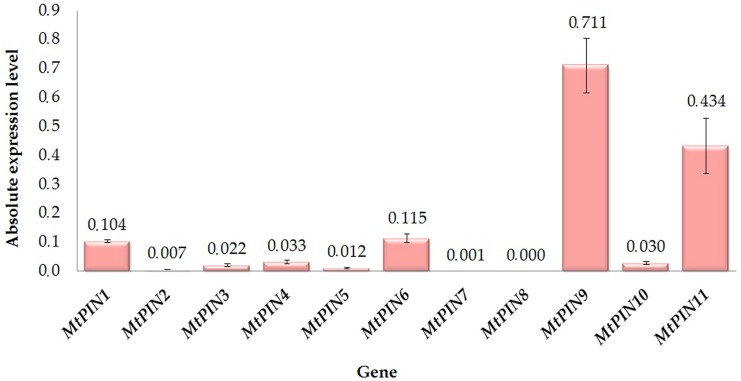
Absolute, normalized level of *PINs* expression in *M. truncatula* root nodules. Mean values (±SE) are derived from two biological replicates for which three individual qPCR reactions were performed (*n* = 6). Expression levels for each *PIN* was normalized to the endogenous control.

The absolute, normalized level of *MtPIN*s expression in root nodules is presented in Figure 2. Similarly to the results obtained for root tips, the abundance of various *PIN* transcripts in root nodules differed significantly. The highest expression level, compared to the other *MtPIN*s, was attributed to *MtPIN9*. Moreover, *MtPIN11*, *MtPIN6* and *MtPIN1* had also relatively high expression in root nodules. Expression of the other *PIN* genes was marginal, besides *MtPIN8*, which was the only *MtPIN* for which transcription was not detected at all.

To compare transcription patterns of *MtPINs* between root nodules and root tips, relative expression quantification was performed. Statistically significant differences were found for all tested *MtPIN* genes. Ten out of eleven *MtPINs* were downregulated in nodules, compared to root tips. The only *PIN* gene that was up-regulated was *MtPIN9* (Table 2).

Although expressed at lower levels in nodules in comparison to roots (Table 2), *MtPIN11*, *MtPIN6* and *MtPIN1* transcript abundance was relatively high in comparison to the remaining *PINs* (Figure 2). These results imply the highest significance of *MtPIN1*, *MtPIN6*, *MtPIN9* and *MtPIN11*, which are the orthologs of Arabidopsis *AtPIN4*, *AtPIN6*, *AtPIN5* and *AtPIN8*, respectively, in the nodulation process in *M. truncatula*.

## 3. Discussion

It has been shown that PIN-formed proteins play the essential role in the root apical meristem (RAM) activity regulation. By restricting the expression of auxin-inducible *PLETHORA* genes (main determinants for root stem cells differentiation) in the basal part of embryo, PINs lead to the initiation of root primordium formation [28].

Our data showed that *MtPIN2* is the most highly expressed *MtPIN* gene in root tips. It has been previously found that *MtPIN2* expression is upregulated in *M. truncatula* RAM [29], which is consistent with our results. In *A. thaliana*, PIN2 plays a pivotal role in the induction of optimal root tip gravitropic response by directional auxins transport from the tip to the root elongation zone [30,31]. Considering such high abundance of MtPIN2 in *M. truncatula* root tips, it can be concluded that its role is parallel to its *A. thaliana* ortholog. *AtPIN3* has been also demonstrated to be expressed in gravity-sensing tissues [32], thus the high expression of its *M. truncatula* ortholog, *MtPIN3*, in root tips seems to be reasonable. Moreover, *A. thaliana PIN4* is highly expressed and involved in the establishment and maintenance of auxins gradient within root tip [33]. Previous phylogenetic studies and our comparison of *A. thaliana* and *M. truncatula* protein sequences allowed to define MtPIN1 to be most closely related to AtPIN4. In our study, *MtPIN1* expression was also relatively high, compared to other *MtPINs* in root tips. High transcription level was also attributed to *MtPIN4*—the ortholog of *AtPIN1*, which is abundantly expressed in vascular tissues [33,34]. Thus, a relatively high expression level of *MtPIN4* in root tips was most likely caused by its ubiquity. Furthermore, *MtPIN9* has been found to be preferentially expressed in the non-meristematic parts of the root [29], which could explain its almost indiscernible expression in root tips. Nevertheless, based on our results proving high expression of *MtPIN9* in indeterminate root nodules, it cannot be excluded that MtPIN9 may play an important role in the nodule meristem functioning.

Additionally, insightful analysis of real-time qPCR results, presented in this study, revealed some interesting facts, especially compared to previous reports. Firstly, our results indicated that, although on a relatively low level, *MtPIN5* transcript was detectable in root tips and nodules, in both examined biological repetitions. A previous study by Schnabel and Frugoli from 2004 suggested that *MtPIN5* is not expressed in plant tissues, since it originated as a result of *MtPIN4* duplication and thus *MtPIN5* expression could be silenced, as the one possible fate of duplicated genes [18].

Moreover, the same study reported that expression of *MtPIN2* was detected in nodulating roots [18]. However, our analysis showed that although *MtPIN2* is highly expressed in root tips, its transcript abundance is very low in mature nodules. Therefore, its contribution in nodulation and meristematic activity maintenance of nodules is uncertain. On the other hand, it should be noted that during our experiment we tested fully differentiated nodules, in which the expression of *MtPIN2* could be already suppressed. There are some data demonstrating *MtPIN2* expression only in the center and outer cortex of nodules 120 hours after inoculation and in the basal part of nodules 12 days after inoculation, but never in the mature root nodules of *M. truncatula*. Additionally, silencing *MtPIN2*, *MtPIN3* or *MtPIN4* by RNAi resulted in lower number of nodules in *M. truncatula* roots compared to control plants [21]. Therefore, it has been suggested that these PINs are involved specifically in the regulation of root nodules development. Therefore, this could be the reason why *MtPIN2*, *MtPIN3* and *MtPIN4* were no longer highly expressed in mature root nodules tissue in our study. Moreover, a recent report has shown that most *MtPIN* and *MtLAX* genes are upregulated in *M. truncatula* roots after *Sinorhizobium meliloti* infection, while downregulated in the shoots. The same genes were downregulated in both shoots and roots in *dmi3* mutant, which is an infection-resistant mutant of *M. truncatula*, suggesting the important role of PINs in nodulation. However, that study examined nodulating roots, not root nodules themselves, thus it is hard to draw conclusions from comparing it to our data [23].

Besides *MtPIN1*, relatively high expression level in root nodules was attributed to *MtPIN6*, *MtPIN9* and *MtPIN11*, which are the orthologs of *A. thaliana AtPIN6*, *AtPIN5* and *AtPIN8*, respectively, and encode proteins belonging to the so-called PIN5 subclade. In *A. thaliana*, PIN proteins from this subclade have been found to localize in the ER [14,15,16,17]. Our results indicated that especially MtPIN9, which is the ortholog of AtPIN5, might be involved in the nodulation in *M. truncatula*. From all *MtPIN* genes, *MtPIN9* was the only one with statistically significant higher expression level in root nodules in comparison to root tips. Proteins belonging to the PIN5 subclade are responsible for auxins transport from the cytosol to ER lumen, where the enzymes of IAA metabolism pathways are located [35]. Auxins inflow to ER lumen is believed to cause a self-regulation of their metabolism. This particular, intracellular transport results in a decrease of auxins cytosolic concentration, and, consequently, in a decrease of their intercellular flow [14]. Since MtPIN6, MtPIN9 and MtPIN11 are homologous to the transporters from *A. thaliana* PIN5 subclade, it can be assumed that their subcellular location is the same. This may indicate the essential role of intracellular homeostasis and auxins’ metabolism in ER compartments in the development and meristematic activity maintenance of *M. truncatula* root nodules.

Furthermore, relatively high expression of *MtPIN11* in nodules might have influenced low transcript abundance of *MtPIN8*. Both of these genes are considered as orthologs of *AtPIN8* and probably encode proteins with the same or similar functions, which may result in decreased expression of one of them.

There is some strong evidence indicating the role of auxins and their transporters in the development of indeterminate-type root nodules. Research conducted in 2005 on *M. truncatula* demonstrated that, in the apical parts of root nodules, de novo IAA synthesis occurs [36]. Moreover, *Medicago sativa* plants treated with polar auxin transport inhibitors: 1-*N*-naphthylphthalamic acid (NPA) or 2,3,5-triiodobenzoic acid (TIBA) were shown to produce pseudonodules—structures lacking rhizobia and ineffective in dinitrogen fixation [20]. Similar experiments conducted on *M. truncatula* plants gave the same results [37].

Interestingly, auxin accumulation in developing *Trifolium repens* nodules was shown to be preceded by inhibition of PAT [38]. Initial inhibition of auxin flow in the earliest stage of nodulation was demonstrated also in the ethylene-insensitive *sickle* mutant of *M.* *truncatula*. However, 24 h after inoculation, the expression of *MtPIN1* and *MtPIN2* in *M. truncatula* *sickle* mutant roots was increased, which led to boosted auxins accumulation just above the infection site and resulted in a significantly higher number of effective nodules, compared to the wild type [39].

It is also possible that flavonoids, acting as PAT regulators, are essential for nodules formation in *M. truncatula*. Flavonoid-deficient roots, with reduced abundance of chalcone synthase (CHS), the key enzyme in flavonoids biosynthesis pathway, demonstrated increased auxin transport. These *M. truncatula* plants were unable to trigger initial reduction of auxin transport and to form root nodules [22].

In 2004, Schnabel and Frugoli identified five *M. truncatula* genes encoding auxin transporter-like proteins (LAX), which are the auxins influx carriers [18]. Previous research revealed that *MtLAX1* is expressed in young, elongating nodule primordia, specifically in their cortical tissues, where the vasculature is formed [40]. Auxins contribution to nodulation of *M. truncatula* was also indicated by the changes in expression of ARFs (Auxin Response Factors) during the response to *S. meliloti* infection [23,24,25]. ARFs are transcription factors in auxins signaling cascades, and binding to auxin response elements (AuxREs) in genes promoter regions. There are 24 characterized *MtARFs*, while AuxREs were found in the promoters of many genes involved in the initiation of nodulation—rhizobial infection response genes [23].

There is also a set of evidence that during symbiosis initiation some phytohormones can be produced by prokaryotic organisms [41]. In a recent study, genetically modified *S. meliloti* bacteria producing a high amount of IAA were tested in *M. sativa* inoculation. The results demonstrated that prokaryotic auxins were still detectable after bacteria endocytosis from infection threads, also in IV zone of root nodules. Moreover, such plants showed a higher number of nodules than *M. sativa* inoculated by wild-type strain, and their transgenic bacteroids had higher expressions of *nifH* gene, encoding subunits of nitrogenase. These plants also showed up to a 73% increase in the shoot fresh weight and more-branched root system [42].

## 4. Experimental Section

### 4.1. Plant Material, Inoculation with Rhizobia and Growth Conditions

*Medicago truncatula* cv. Jemalong A17 seeds were scarified with 96% sulfuric acid (H_2_SO_4_) for 15 min and subsequently washed for five times in sterile water. Seeds were placed in Petri dishes containing 0.8% water agar and stratified for 12 h in the darkness at 4 °C. After stratification, plates were moved to the growth chambers and grown in the following conditions: 16 h photoperiod, photosynthetic photon flux density (PPFD) of 80–100 µE·m^−2^·s^−1^ and temperature 25 °C. Root tips were harvested from two-day-old seedlings. To obtain root nodules, four-day-old seedlings were inoculated with *Sinorhizobium meliloti* strain 1021 culture, which was grown on Tryptone-Yeast (TY) medium until the optical density at 600 nm (OD_600_) of culture was between 0.6 and 0.8. After inoculation, seedlings were placed in pots filled with perlite and watered with nitrogen-free Fahraeus medium [43] containing 60-times diluted inoculum. Pots were covered with transparent foil for 10 days to ensure seedlings high humidity. Plants were grown for 6 weeks in a 16 h photoperiod, PPFD of 110–170 µE·m^−2^·s^−1^ and at 22 °C until fully developed nodules (>1 mm in diameter) were formed. Plants were watered three times per week: twice with distilled water and once with nitrogen-free Fahraeus medium.

### 4.2. RNA Isolation and cDNA Synthesis

RNA was obtained from *M. truncatula* root nodules harvested six weeks after inoculation and, separately, from root tips of two-day-old seedlings with primary root active meristems. The reason for using plant material from different developmental stages was that in six-week-old *M. truncatula* plants, with expanded lateral roots system, the primary root could be delayed in growth and thus difficult to distinguish. Since lateral roots have distinctive root apical meristem (RAM) and geotropism, their PIN distribution can differ from the primary root tip. In order to compare *PIN* expression level in nodules to a reliable reference point, we used primary roots from young seedlings as well-described control. Total RNA isolation was performed using the GeneMATRIX Universal RNA Purification Kit (EUR_X_, Gdańsk, Poland) with the additional step of on-column DNAse I treatment. RNA concentration, purity and integrity was tested by the spectrophotometric method with Nanodrop 2000 (Thermo Scientific, Waltham, MA, USA) and electrophoretic separation in 1% agarose gel. After equalization of RNA concentration, samples were used for cDNA synthesis using High Capacity cDNA Reverse Transcription Kit (Thermo Fisher Scientific, Waltham, MA, USA).

### 4.3. Real-Time Quantitative PCR

Primers used in this study were designed with the help of Primer3 software (Primer3Plus, Free Software Foundation, Inc., Boston, MA, USA) [44] for 11 *MtPIN*s described previously [18,19]. In order to unambiguously identify the phylogenetic relationship between *M. truncatula* and *A. thaliana* orthologs, we used the Basic Local Alignment Search Tool (BLAST) [45] within the NCBI database. BLAST alignment was performed for each MtPIN’s coding DNA sequence (CDS) and full protein sequence with the *A. thaliana* nucleotide or protein database, respectively. For CDSs alignment, we used discontiguous megablast, while, for protein alignment, blastp algorithm. Orthologs were selected according to the highest query cover score. On the basis of this comparison, we identified *A. thaliana* orthologs that are most closely related to the corresponding MtPINs (Table 1). Each primer pair was designed to amplify 60 to 100 nucleotides long fragments of the first exon of particular *MtPIN* sequence. Real-time qPCR was conducted in the 7500 Fast Real-Time PCR System (Applied Biosystems, Waltham, MA, USA) using Power SYBR Green Master Mix (Thermo Fisher Scientific), according to the manufacturer’s instruction. Reaction conditions and primers sequences are presented in Table 3 and Table 4, respectively. Each PIN expression was tested for two biological replicates and three technical repetitions. Gene encoding subunit of *M. truncatula* ribosomal protein S7 (*RPS7b*) was used as the endogenous reference. The specificity of amplified PCR products was verified by melting curve analysis. Statistical analysis of the results was performed using LinRegPCR [26] (calculation of reaction efficiency) and REST2009 [27] (calculation of relative gene expression level and statistical significance of their differences).

## 5. Conclusions

To conclude, our research revealed possible involvement of MtPIN9 transporter in *M. truncatula* nodulation, which was not shown ever before. Furthermore, based on the relatively high expression in root nodules of *MtPINs*, which are the *A. thaliana* orthologs of genes encoding PIN proteins located in the ER, it can be assumed that development and meristematic activity maintenance of *M. truncatula* root nodules may be associated with intracellular homeostasis of auxins level and their metabolism in the ER. Our work further supports the hypothesis that *M. truncatula* nodulation depends on the auxin level. However, further studies are needed to determine auxins role in different developmental stages and particular zones of the nodules. Elucidation of specific auxin transporters functions in nodules formation and maintenance of their meristematic activity should be of great interest in order to fully understand the auxins mode of action during nodulation process.

## Figures and Tables

**Table 1:** The relationship of *M. truncatula* PINs and their *A. thaliana* orthologs according to Schnabel and Frugoli and Peng et al. [18,19] or identified using BLAST search.

*M. truncatula* Gene/Protein	*A. thaliana* Orthologous Gene according to Schnabel and Frugoli and Peng et al.	*A. thaliana* Orthologous Coding DNA Sequences (CDS) Identified Using BLAST Search	*A. thaliana* Orthologous Protein Sequences Identified Using BLAST Search
MtPIN1	*AtPIN3*	*AtPIN4*	AtPIN4
	*AtPIN4*		
	*AtPIN7*		
MtPIN2	*AtPIN2*	*AtPIN2*	AtPIN2
MtPIN3	*AtPIN3*	*AtPIN3*	AtPIN3
	*AtPIN4*		
	*AtPIN7*		
MtPIN4	*AtPIN1*	*AtPIN1*	AtPIN1
MtPIN5	*AtPIN1*	*AtPIN1*	AtPIN1
MtPIN6	*AtPIN6*	*AtPIN6*	AtPIN6
MtPIN7	*AtPIN2*	*AtPIN2*	AtPIN7
		*AtPIN7*	
MtPIN8	*AtPIN8*	*AtPIN8*	AtPIN8
MtPIN9	*AtPIN5*	*AtPIN5*	AtPIN5
MtPIN10	*AtPIN1*	*AtPIN1*	AtPIN1
MtPIN11	*AtPIN8*	*AtPIN8*	AtPIN8

**Table 2:** Comparison of *MtPINs* expression pattern in root nodules relative to root tips. Statistical analysis was conducted in LinRegPCR [26] and REST2009 [27]. *MtRPS7b*, encoding ribosomal protein S7, was used as the endogenous reference gene. “DOWN” or “UP” means that particular *MtPIN*’*s* expression in nodules in comparison to root tips is significantly lower or higher, respectively.

Gene	Type	Reaction Efficiency	Expression	Standard Error	95% Confidence Interval	*p*-Value	Result
*MtRPS7b*	Reference gene	0.8951	1.000				
*MtPIN1*	Target gene	0.7752	0.107	0.090–0.123	0.085–0.137	0.001	DOWN
*MtPIN2*	Target gene	0.9265	0.001	0.001–0.002	0.001–0.002	0.000	DOWN
*MtPIN3*	Target gene	0.9236	0.007	0.006–0.011	0.004–0.012	0.000	DOWN
*MtPIN4*	Target gene	0.9509	0.010	0.007–0.017	0.004–0.023	0.000	DOWN
*MtPIN5*	Target gene	0.9748	0.288	0.194–0.445	0.117–0.576	0.000	DOWN
*MtPIN6*	Target gene	0.9849	0.235	0.151–0.351	0.105–0.440	0.000	DOWN
*MtPIN7*	Target gene	0.8672	0.146	0.098–0.215	0.079–0.300	0.001	DOWN
*MtPIN8*	Target gene	0.7412	0.012	0.009–0.016	0.007–0.019	0.002	DOWN
*MtPIN9*	Target gene	1.0000	29.32	19.258–40.578	15.769–55.179	0.000	UP
*MtPIN10*	Target gene	0.9001	0.044	0.033–0.055	0.023–0.090	0.002	DOWN
*MtPIN11*	Target gene	0.9686	0.445	0.263–0.658	0.179–1.515	0.007	DOWN

**Table 3:** Real-time qPCR conditions.

Temperature	Time
PCR	
50 °C	20 s
95 °C	10 min
40 Cycles:	
95 °C	15 s
60 °C	1 min
Melting curve	
95 °C	15 s
60 °C	1 min
95 °C	30 s
60 °C	15 s

**Table 4:** Accessions of genes and primers sequences used for real-time qPCR. The exact sequence of each *MtPIN* first exon fragment that was used for the primer design is also shown (primers binding sites are marked by shading). Underlines represent start codon of each *PIN* gene and dots (…) represent the discontinuity within the sequence. bp: base pairs.

Gene/ID	Forward (F) and Reverse (R) Primer Sequence	Sequence of Each *MtPIN* First Exon Fragment	Product Length
	5′–3′		(bp)
*MtPIN1* (MTR_4g084870)	F: TCCACTTTACGTAGCCATGATCT	ATGATAACCTGGCACGATCTATACACAGTTTTAACCGCAGTAGTTCCACTTTACGTAGCCATGATCTTAGCCTATGGCTCCGTACGGTGGTGGAAAATATTCTCACCGGACCAATGTTCCGGCATAAACCGTTTCGTCGC	74
	R: AACATTGGTCCGGTGAGAAT		
*MtPIN2* (MTR_4g127100)	F: CGAAGATGAGACATTGAGGATG	ATGATTACCGGTAAGGATATATACGATGTTTTCGCA...CGAAGATGAGACATTGAGGATGCATAAGAAAAGGGGAGGGAGGAGTATGAGTGGTGAGTTGTTCAATAATGGTGGTTCTTACCCTCCTCCAAATCCTATGC	74
	R: CACCATTATTGAACAACTCACCA		
*MtPIN3* (MTR_1g030890)	F: CTGGCCTCAACGTGTTCC	ATGATAACACTAAAAGATCTTTACACTGTCTTAACAGCAGTGGTTCCA...CTGGCCTCAACGTGTTCCGAAATTCGGAACAATCGGAAGAGGGTGCTAAGGAGATCAGGATGGTGGTGGCTGATGAACATAATCAAAA	68
	R: CACCACCATCCTGATCTCCT		
*MtPIN4* (MTR_6g069510)	F: TGGTGCCACTTTATGTAGCTATG	ATGATCACTTTAACAGATTTCTACCATGTCATGACAGCAATGGTGCCACTTTATGTAGCTATGATCTTAGCTTATGGATCAGTAAAATGGTGGAAAATATTTTCACCTGATCAATGTTCAGGAATCAACCGTTTTGTTGCA	92
	R: ACGGTTGATTCCTGAACATTG		
*MtPIN5* (MTR_8g107360)	F: CGTGGCTATGATATTAGCTTATGG	ATGATAACGTTAACAGATTTCTACCATGTGATGACATCAATGGTGCCACTTTACGTGGCTATGATATTAGCTTATGGTTCAGTGAAATGGTGGAAGATATTCTCTCCCGATCAATGCTCCGGCATCAATCGCTTCGTTGCT	66
	R: GAGCATTGATCGGGAGAGAA		
*MtPIN6* (MTR_1g029190)	F: TAAACCGATTCGTCGCAGTT	ATGGTGACAAGAGAAGATTTATACAACGTGATGTGTGCAATGGTACCTC...TAAACCGATTCGTCGCAGTTTTTGCTGTTCCAGTTCTATCTTTTCACTTCATTTCTCTCAACAATCCTTATCAAATGGACACAAAATTTAT	67
	R: GGATTGTTGAGAGAAATGAAGTGA		
*MtPIN7* (MTR_4g127090)	F: TTGTGCCACTATATGTCGCTATG	ATGATTACCGGCAAGGACATATACAATGTTTTAGCGGCGATTGTGCCACTATATGTCGCTATGATATTAGCATATGGTTCGGTCCGATGGTGGAAAATCTTCACACCAGATCAATGTTCTGGAATAAACCGTTTTGTCTC	94
	R: AAACGGTTTATTCCAGAACATTG		
*MtPIN8* (MTR_7g009370)	F: TTTCCTTAGCCAATGTTTATCATGT	ATGATTTCCTTAGCCAATGTTTATCATGTAATAACAACAACTGTCCCATTATATGTAACAATGATACTAGCCTATGTCTCAGTGAAATGGTTTAAGATCTTCACACAAGAACAATGTTCAGGAATAAACAAATTTGTTGC	95
	R: GATCTTAAACCATTTCACTGAGACATA		
*MtPIN9* (MTR_7g079720)	F: AGCAGTGGTGCCACTCTATTTT	ATGATTGGGTGGGAAGACGTGTACAAAGTTATTGTAGCAGTGGTGCCACTCTATTTTGCACTAATATTAGGCTATGGTTCTGTAAGGTGGTGGAAAATTTTCACAAGAGAACAATGTGATGCAATAAACAAACTAGTTT	96
	R: TTGTTTATTGCATCACATTGTTCTC		
*MtPIN10* (MTR_7g089360)	F: TGGTGTTGCTAAAGCTAATGGA	ATGATAAGTGCTTTAGACTTATACCATGTCCTCACAGCAGTAGTACC...TGGTGTTGCTAAAGCTAATGGAAATGGTGGAAATGGCTACCCTGCTCCTCATAGTGCAGGGATTTTTTCACCTGTGGCTAATAAGAAAAA	61
	R: CCCTGCACTATGAGGAGCA		
*MtPIN11* (MTR_6g011400)	F: ACAGCCACTGTCCCATTATATGT	ATGATTTCCTTAATTGATGTCTATCATGTAGTAACAGCCACTGTCCCATTATATGTAACTATGTTACTAGCATACATTTGTGTTAAATGGTGGAAACTTTTCACACCAGATCAATGTGCAGGCATAAACAAATTTGTAGC	87
	R: TGCACATTGATCTGGTGTGA		
*MtRPS7b* (MTR_8g087480)	F: GAAACAACACTGCAATTTACAGGA	ATGACCTTACCATACCCATACCATCACCATTGTTGT...GAAACAACACTGCAATTTACAGGAAACTATCAGGCAAAGATGTTGTCTTTAGTATCCCGTTACTGAGGCTTAGGTTCTGTTTCTCAATTTTGATTTTGTTTCATG	74
	R: CCTAAGCCTCAGTAACGGGATA		

## Abbreviations

ATP	adenosine triphosphate
AUX1	AUXIN1
BLAST	basic local alignment search tool
bp	base pair
CDS	coding DNA sequence
cv.	cultivar
ER	endoplasmic reticulum
IAA^−^	anionic indole-3-acetic acid
IAAH	protonated form of IAA
LAX	LIKE AUX1
NCBI	national center for biotechnology information
PAT	polar auxin transport
PCR	polymerase chain reaction
PGP	P-glycoprotein
qPCR	quantitative polymerase chain reaction
RAM	root apical meristem
SE	standard error
